# Fabrication and Characterization of Graphene Microcrystal Prepared from Lignin Refined from Sugarcane Bagasse

**DOI:** 10.3390/nano8080565

**Published:** 2018-07-24

**Authors:** Pei-Duo Tang, Qi-Shi Du, Da-Peng Li, Jun Dai, Yan-Ming Li, Fang-Li Du, Si-Yu Long, Neng-Zhong Xie, Qing-Yan Wang, Ri-Bo Huang

**Affiliations:** 1 State key Laboratory of Bioenergy Enzyme Technology, National Engineering Research Center for Non-Food Biorefinery, Guangxi Academy of Sciences, Nanning 530007, China; tangpeiduo@gxas.cn (P.-D.T.); 18577113352@126.com (J.D.); lym810555@163.com (Y.-M.L.); dfl@gxas.cn (F.-L.D.); xbslongsiyu@163.com (S.-Y.L.); xienengzhong@gxas.cn (N.-Z.X.); qingyanw@126.com (Q.-Y.W.); 2 Gordon Life Science Institute, 53 South Cottage Road, Belmont, MA 02478, USA; 3 Institute of Surface Micro and Nano Materials, Xuchang University, Xuchang 461000, China; lidapengabc@126.com

**Keywords:** graphene microcrystal, glassy carbon, lignin, bagasse, biorefinery, graphene, biomass

## Abstract

Graphene microcrystal (GMC) is a type of glassy carbon fabricated from lignin, in which the microcrystals of graphene are chemically bonded by sp^3^ carbon atoms, forming a glass-like microcrystal structure. The lignin is refined from sugarcane bagasse using an ethanol-based organosolv technique which is used for the fabrication of GMC by two technical schemes: The pyrolysis reaction of lignin in a tubular furnace at atmospheric pressure; and the hydrothermal carbonization (HTC) of lignin at lower temperature, followed by pyrolysis at higher temperature. The existence of graphene nanofragments in GMC is proven by Raman spectra and XRD patterns; the ratio of sp^2^ carbon atoms to sp^3^ carbon atoms is demonstrated by XPS spectra; and the microcrystal structure is observed in the high-resolution transmission electron microscope (HRTEM) images. Temperature and pressure have an important impact on the quality of GMC samples. With the elevation of temperature, the fraction of carbon increases, while the fraction of oxygen decreases, and the ratio of sp^2^ to sp^3^ carbon atoms increases. In contrast to the pyrolysis techniques, the HTC technique needs lower temperatures because of the high vapor pressure of water. In general, with the help of biorefinery, the biomass material, lignin, is found to be qualified and sustainable material for the manufacture of GMC. Lignin acts as a renewable substitute for the traditional raw materials of glassy carbon, copolymer resins of phenol formaldehyde, and furfuryl alcohol-phenol.

## 1. Introduction

Glassy carbon, or vitreous carbon, is a non-graphitizing and crystallized carbon, which combines glassy and ceramic properties with those of graphite, but cannot be graphitized at any temperature [1,2,3]. Glassy carbon is not amorphous matter, but has a glass-like microcrystal structure, consisting of microcrystals of fullerene, carbon nanotube, and/or graphene [1,2,3]. In the microstructure of glassy carbon materials, the sp^2^ carbon atoms are chemically bonded by sp^3^ carbon atoms, forming a 3D structural network [4,5,6,7,8,9]. The possession of such a unique structure grants glassy carbon a great variety of physical and chemical properties [10,11,12,13,14]. The most important properties of glassy carbon are high hardness, low density, high temperature resistance, low electrical and thermal resistance, low friction, extreme impermeability to gases and liquids at very high temperatures, and resistance to chemical attack [10,15,16,17]. Glassy carbon materials are widely used in many extreme fields because of their unique and excellent properties.

Typically, glassy carbon is manufactured in an inert gaseous atmosphere by heat treatment at elevated temperatures (1000–2400 °C) from organic polymeric precursors, such as copolymer resins of phenol formaldehyde or furfuryl alcohol-phenol [8,18,19]. These polymers are used because of their high carbon yield upon pyrolysis, in which the increase in the proportion of carbon after carbonization is around ~50% compared to before [20]. Although glassy carbon was first discovered in the 1960s [1,21], the fabrication methods of glassy carbon have been continuously improved upon. Theoretical study on the structure of glassy carbon has also steadily progressed since its discovery. In recent years, the newly invented fullerene, carbon nanotube, and graphene helped researchers to restudy and re-evaluate the microstructures of glassy carbon [7,8,22,23]. Graphene, particularly, has obtained extended uses in drug delivery, membrane, biomedical and adsorption applications, in addition to use in sensors [24,25,26,27,28].

In a recent study, it was found that by adding a small fraction of carbon nanotubes to a phenol-formaldehyde polymer, similar glassy carbon could be achieved at 800 °C, 200 °C lower than the previous temperature [7]. Another study reported that compression of glassy carbon at high pressure and certain temperatures induced the local buckling of graphene sheets through sp^3^ nodes to form interpenetrating graphene networks with a long-range disorder and short-range order on a nanometer scale [10]. Such compressed glassy carbons have extraordinary specific compressive strengths—more than two times that of commonly used ceramics—and simultaneously exhibit robust elastic recovery in response to local deformations [10]. A study reported that in the fabrication of glassy carbon, the traditional raw materials, phenol formaldehyde or furfuryl alcohol-phenol, were successfully replaced by camphor, a natural and renewable source [29].

The traditional precursors of glassy carbon are produced from coal, petroleum, or natural gas [3,4,19,30]. The over-consumption of fossil materials has caused global warming, frequent occurrences of extreme weather, and ecological disaster [31,32]. To deal with the global environmental and ecological problems, biomass (particularly the waste from the agriculture and forest industries), is the best way to replace coal, petroleum, and natural gas as both the energy fuel and raw materials of the chemical and material industries [33,34,35,36,37,38]. Lignin is the second largest renewable biomass material in the natural world and has been used in the fabrication of graphene by several authors [39,40]. In this study, the green and sustainable biomass material, lignin, is used to prepare the graphene microcrystal, a type of glassy carbon, in which the nanoscale graphene fragments are chemically bonded by sp^3^ carbon atoms. In this study, such glassy carbon is named graphene microcrystal (GMC), to differentiate it from other types of glassy carbon [1,2,3,6,9].

## 2. Experimental Section

Not all commercially available lignin products are good materials for the fabrication of GMC. The Kraft lignin [41], is a sulfate lignin, i.e., lignin containing sulfur, which is not suitable for the fabrication of GMC. In this study, the precursor lignin is refined, using an organosolv technique, from sugarcane bagasse, which is a pure lignin polymer, consisting solely of carbon, oxygen, and hydrogen.

### 2.1. Biorefinery of Sugarcane Bagasse

The fresh sugarcane bagasse used in this study is provided by a cane mill of Nanning sugar industry CO., LTD (http://www.nnsugar.com/) in Guangxi, China. The main components in sugarcane bagasse are comprehensively analyzed, and the general results are as follows: Cellulose 45–55%, hemicellulose 20–25%, lignin 18–24%, ash 1–4%, and waxes <1%, which are consistent with the reference [42].

**Figure 1:**
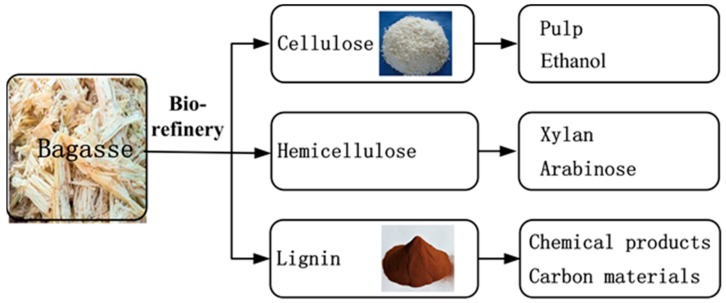
The sugarcane bagasse is refined into cellulose, hemicellulose, and lignin using an ethanol-based organosolv technique. The cellulose is used for the production of pulp and ethanol fuel, the hemicellulose is used for the medicinal and nutritional product xylan, and the lignin is used for the fabrication of graphene microcrystals (GMC) described in this study.

The ethanol-based organosolv technique, developed by Guangxi Botanical Institute (http://www.gxib.cn/), is used for the biorefinery of bagasse. The bagasse is ground to a 40 mesh (0.45 mm) powder, followed by drying in a loft drier for 2 h at 120 °C. The bagasse powder is then soaked in a 55 wt% ethanol-water solution in pH = 3.0~4.0 conditions, followed by heating to 200 °C for 100 min in a specially designed boiler. In this process, the chemical bonds between lignin and cellulose/hemicellulose are broken, and lignin dissolves in the ethanol-water solution. The undissolved cellulose and hemicellulose are removed from the solution to a reactor, where the hemicellulose is hydrolyzed to xylose at 140 °C for 20 min in acidic conditions, after which it is separated with cellulose. The dissolved lignin in the ethanol-water solution is recovered by solvent vaporization. Unlike Kraft lignin [43,44], refined lignin has the same structure as pure lignin, consisting of only carbon, oxygen and hydrogen elements, with no other heteroatoms, such as sulfur, nitrogen, and sodium. The technical scheme of the biorefinery is illustrated in Figure 1.

### 2.2. Structure of Lignin

**Figure 2:**
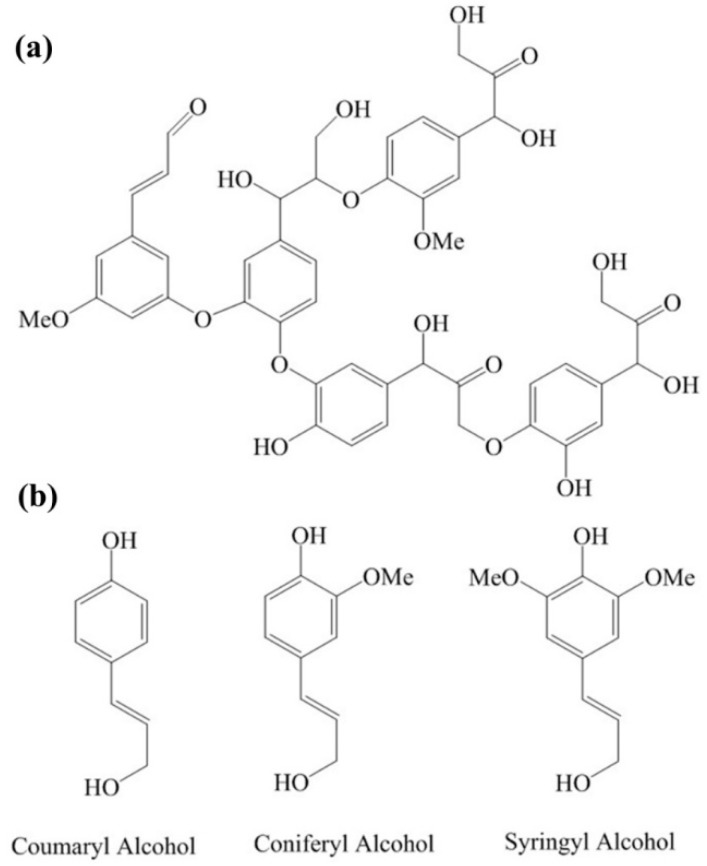
The chemical structure and composition of lignin. (**a**) Structural fragment of a lignin polymer. Lignin is a series of randomly cross-linked phenolic polymers. It has no specific chemical formulation, and this differs from species to species. (**b**) The chemical structures of three monomers of lignin: *p*-coumaryl alcohol, coniferyl alcohol, and sinapyl alcohol, respectively.

The chemical composition of lignin is shown in Figure 2, where (a) is the structural fragment of lignin polymer; and (b) shows the chemical structures of three composition monomers of lignin: *p*-Coumaryl alcohol, coniferyl alcohol, and sinapyl alcohol, respectively [45,46]. The chemical structure of lignin, shown in Figure 2a, is an approximate description, as the composition of lignin varies from species to species. Lignin has the most abundant carbon component among all natural organic polymers, in which the element composition is around 63.4% carbon, 30% oxygen, 5.9% hydrogen, and 0.7% ash (mineral components) [47], corresponding, approximately, to the formula (C_31_H_34_O_11_)n. In lignin, the carbon element is higher than that in the traditional precursor of glassy carbon, phenol formaldehyde, and furfuryl alcohol-phenol. Particularly, lignin contains both sp^2^ and sp^3^ carbon atoms at a ratio of approximately 33:12, which is very favorable for the composition of glassy carbon [10,48]. Lignin is, in fact, the precursor of phenol formaldehyde and furfuryl alcohol-phenol [46,49], the traditional raw materials of glassy carbon.

**Figure 3:**
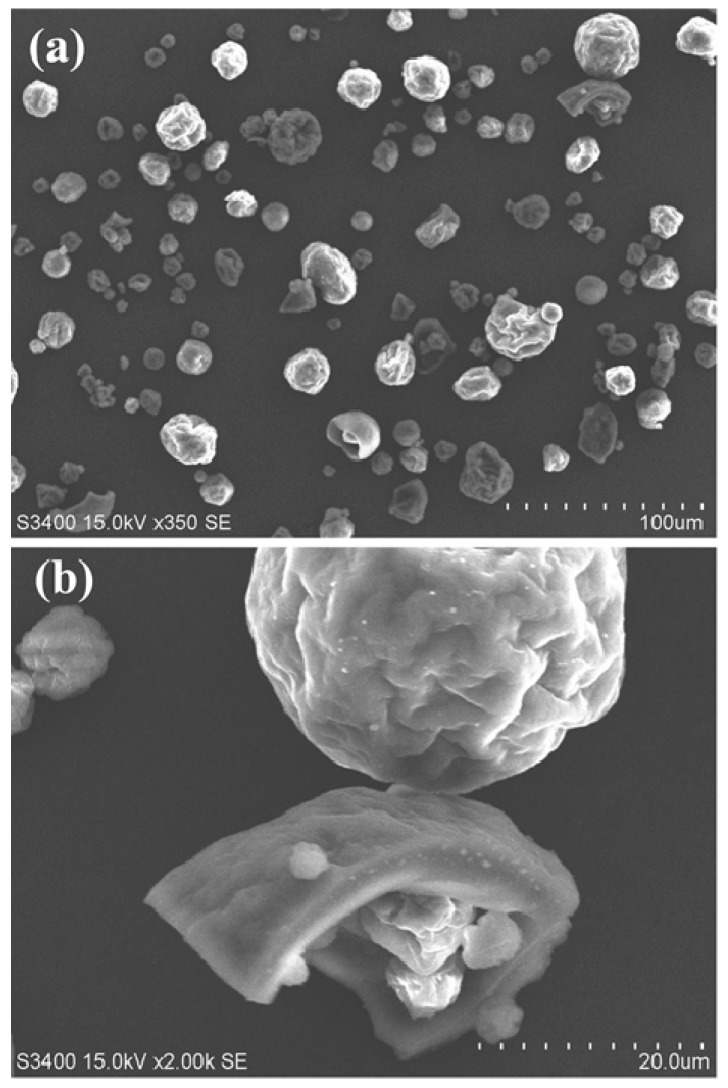
The SEM (scanning electron microscope) images of lignin particles obtained from an ethanol-water solution by evaporation of the solvent. (**a**) Most lignin particles are rough spheres with a diameter of tens to hundreds of micrometers. (**b**) A close view of larger lignin particles. Some of the lignin particles may be hollow spheres or pieces of broken spheres.

Figure 3 shows the SEM images of lignin particles obtained from an ethanol-water solution by evaporation of the solvent. Most lignin particles are rough spheres with a diameter of tens to hundreds of micrometers. Some of the lignin particles may be hollow spheres or pieces of broken spheres.

### 2.3. Fabrication of GMC

Two technical schemes are used in the preparation of GMC samples from lignin. Scheme 1 is a one-step procedure, in which the pyrolysis of lignin is performed in a tubular furnace under a nitrogen atmosphere with a temperature control program. The physical and chemical properties of the glassy carbon samples are controllably influenced by the heating procedure [7,8]. The temperature control program is carefully designed as follows. Period 1 is a melting process of the lignin powder, in which the temperature increases from room temperature to 180 °C with a ramp rate of 1 °C min^−1^ under a flow of nitrogen, maintaining the temperature at 180 °C for 1 h for complete and uniform melting. Period 2 is a thermal pyrolysis process of lignin in the temperature range of 180 °C to 450 °C with a ramp rate of 1 °C min^−1^, in which the volatile organic molecules and water molecules escape with the nitrogen flow. Period 3 is the carbonization and graphitization of lignin at high temperature, in which the temperature increases from 450 °C to a chosen high temperature (800 °C, 1000 °C, or 1200 °C) at a ramp rate of 2 °C min^−1^ in a nitrogen atmosphere, maintaining the high temperature for 3 h. Period 4 is an annealing process of glassy carbon in a nitrogen atmosphere. In this step, the temperature decreases from the chosen high temperature to 30 °C at a ramp-up rate of 5 °C min^−1^.

**Figure 4:**
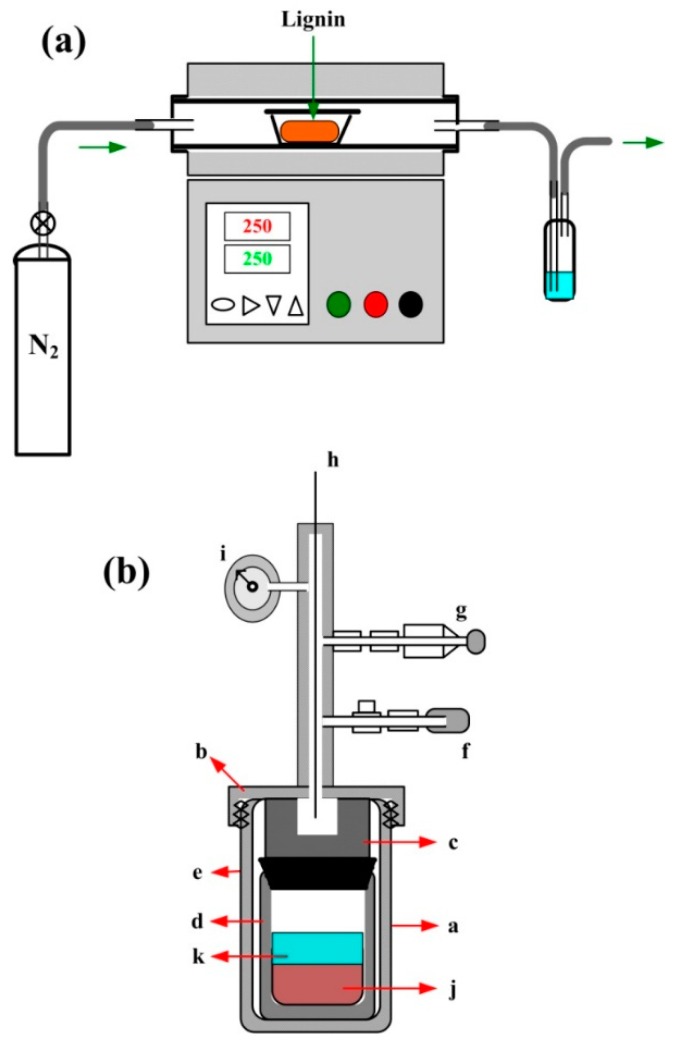
Two technical schemes used in the preparation of GMC samples. (**a**) The lignin pyrolysis procedure in a tubular furnace with a temperature control program under a nitrogen atmosphere and at atmospheric pressure. (**b**) The hydrothermal carbonization (HTC) procedure of lignin at 240 °C and the corresponding vapor pressure of water, approximately 100 mPa. (a: Cylinder; b: Cover; c: Block; d: Teflon cylinder; e: Teflon cover; f: Relief valve; g: Safety valve; h: Thermocouple; i: Pressure gage.)

Scheme 2 is a two-step procedure that combines the hydrothermal carbonization (HTC) [50,51,52,53] with the pyrolysis of lignin. In the first step, 10 g lignin and 50 g deionized water are sealed in a 100 ml HTC reactor, where an aquathermolysis reaction of lignin takes place at 240 °C and 100 mPa, the vapor pressure of water corresponding to that temperature. In the second step, the pyrolysis reaction of partially carbonized lignin is performed in a tubular furnace at a high temperature, as in Scheme 1. The experimental equipment of Scheme 1 and Scheme 2 are illustrated in Figure 4.

## 3. Results and Discussion

**Figure 5:**
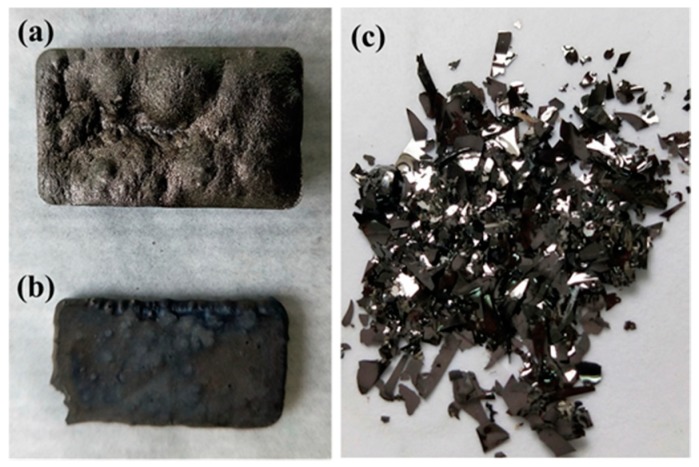
The photographs of GMC samples prepared in this study. (**a**) The GMC sample prepared at 800 °C in a tubular furnace. (**b**) The GMC sample prepared at 1200 °C in a tubular furnace. (**c**) The GMC sample prepared using an HTC-Pyr two-step technique at 240 °C and 800 °C.

In this section, the GMC samples prepared using the two technical schemes under different reaction conditions are characterized using SEM and high-resolution transmission electron microscope (HRTEM) images and spectra of FTIR, XRD, Raman, and XPS. The photographs of typical GMC samples prepared in this study are shown in Figure 5.

### 3.1. GMC Samples Prepared Using Scheme 1

The GMC samples, prepared using a pyrolysis procedure, are described and characterized as follows.

#### 3.1.1. SEM and TEM Images of GMC Samples

**Figure 6:**
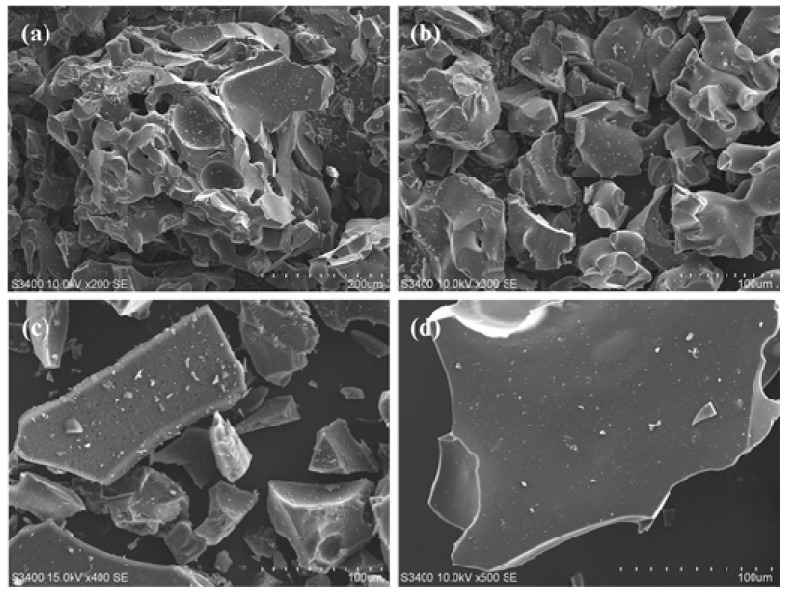
The SEM images of GMC samples. (**a**) GMC sample prepared in a tubular furnace at 800 °C. (**b**) GMC sample prepared in a tubular furnace at 1000 °C. (**c**) GMC sample prepared in a tubular furnace at 1200 °C. (**d**) A close view of a GMC sample prepared in a tubular furnace at 1200 °C. In the GMC sample, prepared at 800 °C, there are more holes. However, with the temperature increase the GMC samples become more compact and uniform.

**Figure 7:**
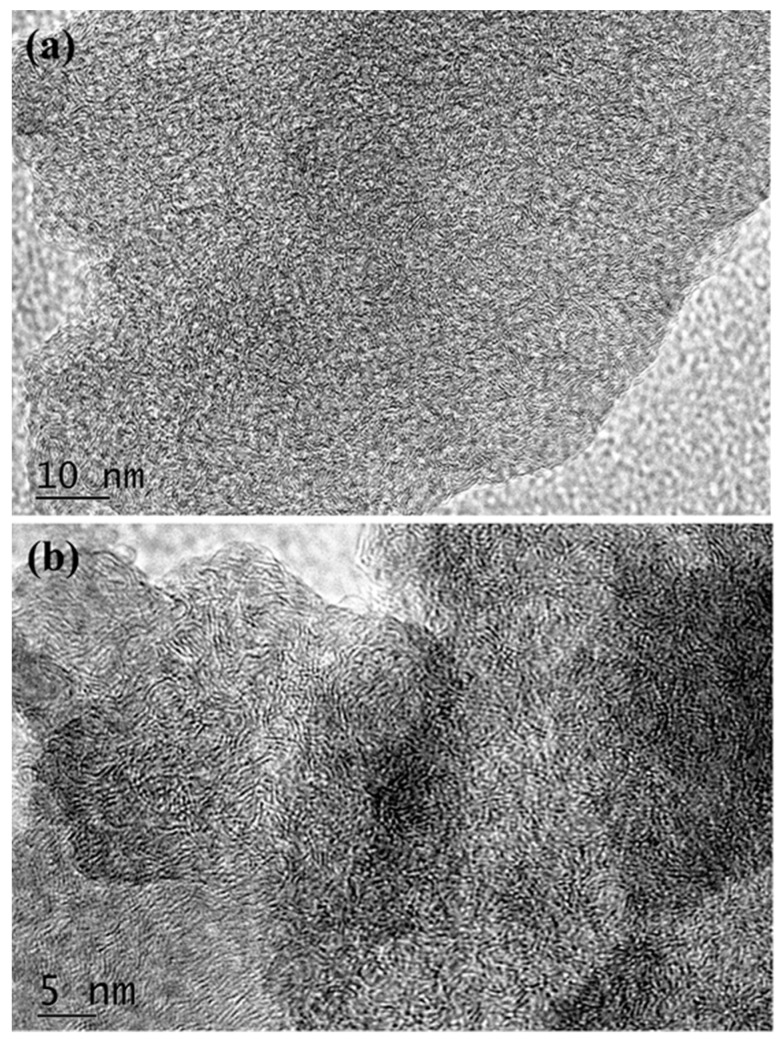
The HRTEM (high resolution transmission electron microscope) images of the GMC samples. (**a**) The TEM image of GMC sample at a resolution of 10 nm. (**b**) The TEM image of a GMC sample at a resolution of 5 nm. The TEM images clearly show the graphene sheets and the glassy-like microcrystals in the GMC samples. In GMC samples, most graphene fragments consist of 3 to 5 graphene sheets with dimensions of 3 to 5 nm.

The hard and rigid graphene microcrystal samples are ground into powders. The SEM images of glassy carbon powders, prepared using Scheme 1 at 800 °C, 1000 °C and 1200 °C, are shown in Figure 6a–c, respectively. In Figure 6, the broken slags of GMC samples show sharp edges and curved faces, very similar to the powder of common glasses and ceramics. In the GMC sample prepared at 800 °C, shown in Figure 6a, there are many holes. However, with the temperature increase, the GMC samples become more compact and uniform. The HRTEM (high-resolution transmission electron microscope) images of the GMC samples in Figure 7a,b clearly show the glassy-like microcrystal structures and the crystal fragments of the graphene sheets. In the GMC samples, most graphene fragments consist of three to five parallel graphene sheets at a size of 3 to 5 nm.

#### 3.1.2. FTIR Spectra of Lignin and GMC Sample

**Figure 8:**
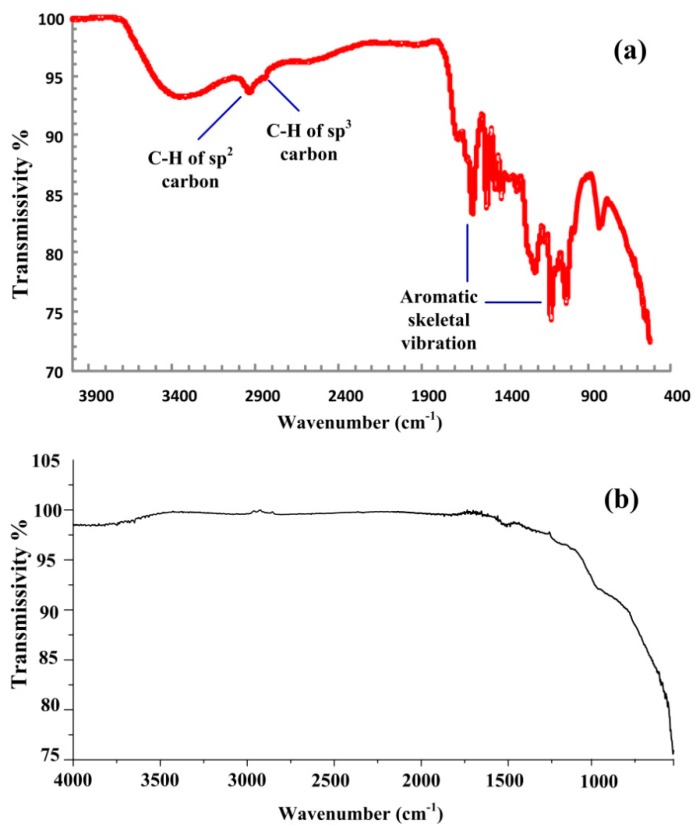
The FTIR spectra of lignin and a GMC sample. (**a**) The FTIR spectrum of lignin (red) isolated and purified from sugarcane bagasse. (**b**) The FTIR spectrum of a GMC sample prepared at 1000 °C. The peaks of C-H bonds and aromatic bonds almost disappear in the FTIR spectrum of the GMC sample, indicating the full carbonization of the precursor lignin during the thermal pyrolyzation reaction.

For comparison, the FTIR spectra of the precursor lignin and the GMC sample prepared at 1000 °C are shown in Figure 8a,b, respectively. In Figure 8a, the two peak bands around 1400–1600 cm^−1^ and 1000–1200 cm^−1^ are the vibrations of aromatic skeletal bonds [54]. The two peak bands in the range 2950–3050 and 2850–2945 cm^−1^ correspond to the C-H bond stretching vibrations of sp^2^ and sp^3^ carbon atoms, respectively [29]. Based on the peak intensity analysis, the ratio of sp^2^ to sp^3^ carbon atoms in lignin is calculated to be 3.84 [29]. In contrast, all above peaks of C-H bonds and aromatic bonds almost disappear in the FTIR spectrum of a GMC sample, indicating the full carbonization of the precursor lignin during the thermal pyrolyzation reaction.

#### 3.1.3. XRD Patterns of GMC Samples

**Figure 9:**
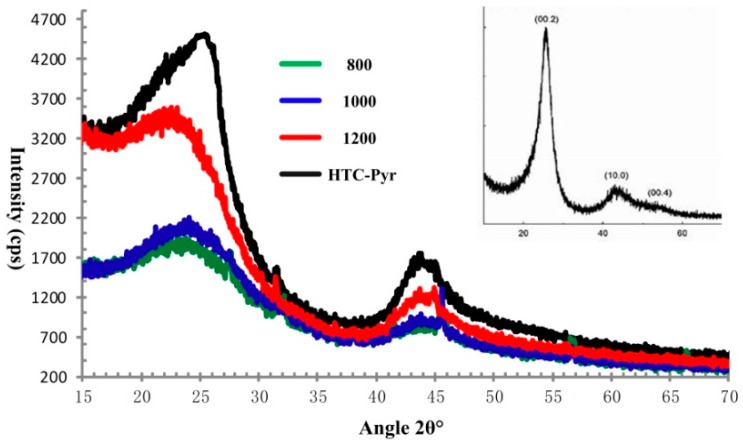
The XRD patterns of GMC samples, prepared at three temperatures: 800 °C (green), 1000 °C (blue), 1200 °C (red), and using an HTC-Pyr (black) technique. The XRD patterns of GMC samples are very close to the XRD pattern (inset) of reduced graphene oxide (rGO) (www.nanonics.co.il), indicating the presence of an oxygen group in the graphene sheets of the GMC samples.

X-ray diffraction (XRD) is typically used to characterize the structure and layers of graphene materials [55,56]. The XRD patterns of GMC samples, prepared at 800 °C, 1000 °C, and 1200 °C are shown in Figure 9. The diffraction peak centered around 2θ = 25°~26.5° comes from the reflection of the 002 planes in the GMC samples. In Figure 9, with the temperature increase, the diffraction peak of the 002 plane moves from 25° to 26.5° and increases in intensity. The value of 26.5° corresponds to the interlayer space, 0.34 nm, in the graphene crystal, indicating superior graphitization of GMC samples under higher temperatures. In Figure 9, the XRD pattern of reduced graphene oxide (rGO), cited from Nanonics Imaging Ltd. (www.nanonics.co.il) is given in the inset to the figure. The XRD patterns of the GMC samples are highly similar to the XRD pattern of rGO, indicating the presence of oxygen groups in the graphene sheets of the GMC samples.

#### 3.1.4. Raman Spectra of GMC Samples

**Figure 10:**
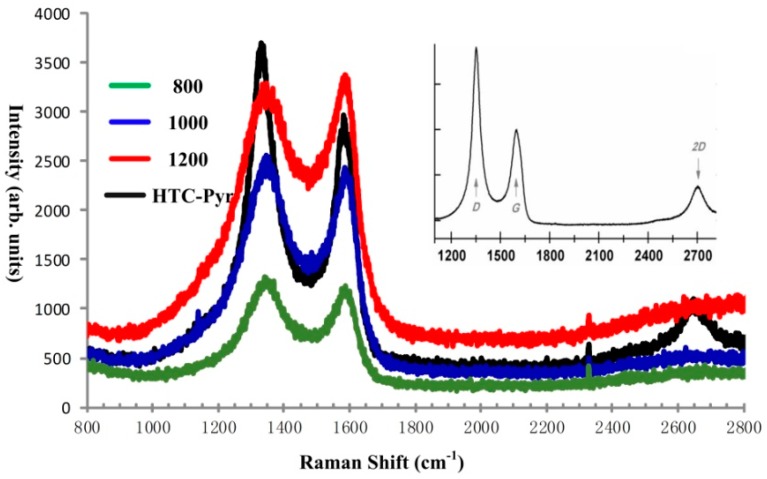
The Raman spectra of GMC samples prepared at four temperatures: 800 °C (green), 1000 °C (blue), 1200 °C (red), and using an HTC-Pyr (black) technique. The Raman spectra of GMC samples are very similar to the Raman spectrum (see inset) of rGO (www.nanonics.co.il), indicating the presence of graphene sheets in the GMC samples. However, the oxygen fraction in GMC samples is less than that in rGO.

A Raman spectrum is a useful tool for the characterization of graphene samples and related materials [57,58,59]. The Raman spectra of GMC samples prepared at three temperatures (800 °C, 1000 °C, 1200 °C) are shown in Figure 10, in which there is a high D band at 1339 cm^−1^, a relatively lower G band at 15,917 cm^−1^, and a flat 2D band at 2655 cm^−1^. Comparing the Raman shifts of the GMC samples in Figure 10, prepared under different temperatures, as the temperature increases the intensities of peaks D and G get higher. The spectrum on the upper right corner in Figure 10, is the typical Raman shift of reduced graphene oxide (rGO), cited from Nanonics Imaging Ltd. (www.nanonics.co.il). The Raman shifts of GMC samples are very similar to those of rGO, indicating the existence of oxygen groups in graphene sheets of the GMC samples. However, the oxygen fraction in GMC samples is less than that in rGO.

#### 3.1.5. XPS Spectra of GMC Samples

**Figure 11:**
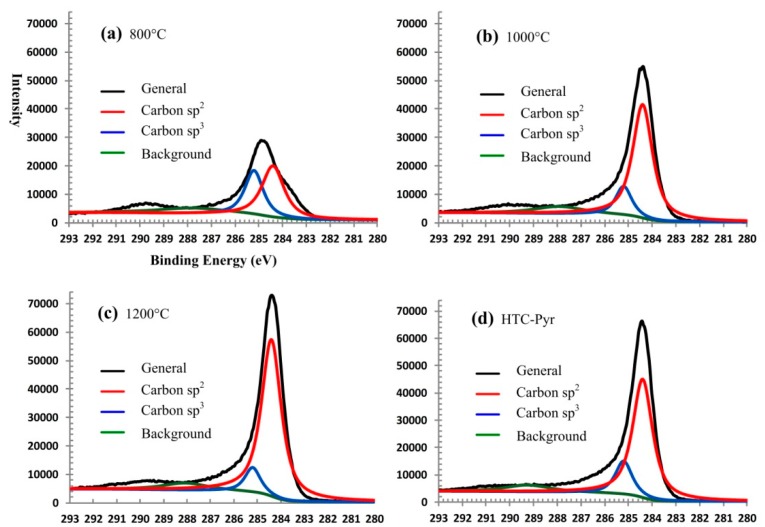
The XPS spectra of GMC samples prepared at three temperatures and using an HTC-Pyr technique. (**a**) XPS spectra of GMC samples prepared at 800 °C. (**b**) XPS spectra of GMC samples prepared at 1000 °C. (**c**) XPS spectra of GMC samples prepared at 1200 °C. (**d**) XPS spectra of GMC samples prepared using HTC-Pyr method. The carbon C1s profiles are separated into carbon sp^2^ peaks (red, centered at 284.4 eV) and carbon sp^3^ peaks (blue, centered at 285.2 eV) using the XPS peak-fitting program, XPSPEAK 4.1.

The XPS spectra [60,61,62] of GMC samples prepared at three temperatures (800 °C, 1000 °C, and 1200 °C) are shown in Figure 11a–c, respectively, and the experimental data from XPS experiments are reported in Table 1. In the XPS spectra, the carbon C1s profiles are separated into carbon sp^2^ peaks (red, centered at 284.4 eV) and carbon sp^3^ peaks (blue, centered at 285.2 eV) [63] using the XPS peak fitting program XPSPEAK 4.1 (http://xpspeak.software.informer.com/4.1/). An interesting phenomenon was observed in which a temperature increase caused the sp^3^ peak (blue) to diminish, while the sp^2^ peak (red) increased in intensity, implying the graphitization of GMC sample increases with the elevation of temperature. In Table 1, the ratio of sp^2^ carbon atoms to sp^3^ carbon atoms is 8:1 in the GMC sample prepared at 1200 °C. In the graphene microcrystal samples, oxygen is the main impurity. With the elevation of temperature, the fraction of oxygen decreases, while the fraction of carbon increases. When the temperature increases to 1200 °C, the fraction of carbon reaches 93.27%, while the fraction of oxygen decreases to 6.27%.

### 3.2. GMC Sample Prepared from Scheme 2

The GMC sample, prepared using technique Scheme 2 (HTC and Pyrolysis), is described and characterized in this section. The big difference between Scheme 1 and Scheme 2 is that in Scheme 1 each reaction was carried out under atmospheric pressure (0.1 mPa), while in Scheme 2 the HTC reaction takes place under higher pressure, around 100 mPa, the vapor pressure of water at 240 °C. However, in the HTC reaction, the temperature cannot be elevated too high for safety reasons. In the second step, the partially carbonized lignin is further graphitized in a tubular furnace at 800 °C in a nitrogen atmosphere. The photographs and spectra of XRD, Raman, and XPS of GMC samples prepared using Scheme 2 are shown in Figure 5, Figure 9, Figure 10 and Figure 11, respectively, with those of GMC samples prepared using Scheme 1 for comparison. In Figure 5, the photograph of HTC-Pyr graphene microcrystals shows them to be shining and bright due to the flushing of water vapor at high pressure. In Figure 9, the XRD pattern (black) of HTC-Pyr glassy carbon is very close to that of reduced graphene oxide (rGO). In Figure 10, the Raman spectrum that is most similar to reduced graphene oxide (rGO) is that of HTC-Pyr GMC (black). In Figure 11, the ratio of sp^2^ carbon atoms to sp^3^ carbon atoms in HTC-Pyr graphene microcrystals is higher than that of the GMC sample prepared under 1000 °C. In Table 1, the HTC-Pyr GMC possesses a higher fraction of carbon and a lower fraction of oxygen than the GMC sample prepared under 1000 °C.

## 4. Conclusions

Based on this study, some useful conclusions are summarized as follows. (1) Graphene microcrystal (GMC), a type of glassy carbon, is successfully fabricated from lignin, which consists of nanoscale graphene microcrystal fragments, chemically bonded by sp^3^ carbon atoms, forming a glass-like long-range disorder and short-range order microcrystal structure that is demonstrated by XRD, Raman, XPS, SEM, and HRTEM experiments. (2) Lignin, the second-largest sustainable biomass material in the natural world, is proven as a qualified and sustainable material for the manufacture of GMC. Lignin is a substitute for the traditional raw materials of glassy carbon, copolymer resins of phenol formaldehyde, and furfuryl alcohol-phenol. (3) The merit of the organosolv biorefinery technique is that the lignin is isolated and dissolved from lignocellulose, keeping its original chemical structure and with no other chemical elements introduced, unlike Kraft lignin and alkaline lignin. (4) The temperature and pressure have important impacts on the quality of GMC samples. With the elevation of temperature, the ratio of sp^2^ carbon atoms to sp^3^ carbon atoms and the component of carbon increase, while the component of oxygen decreases. The (HTC-pyrolysis) two-step technique could produce a better GMC sample at lower temperatures because of the high vapor pressure in the HTC reactor.

Currently, as-prepared GMC samples contain certain oxygen groups; the graphene fragments are similar to those in rGO. However, upon temperature elevation, the fraction of oxygen decreases. To obtain a better quality of GMC, more experiments must be performed.

## Figures and Tables

**Table 1:** The sp^2^ to sp^3^ ratio of carbon atoms and the elemental ratio in graphene microcrystal samples.

Temperature (°C)	Valence Ratio (%)		Element (%)		
	C-sp^2^	C-sp^3^	Carbon	Oxygen	Other
800	58.05	41.95	79.02	14.95	6.03
1000	77.08	22.92	83.82	12.10	4.08
1200	88.73	11.27	93.27	6.27	0.46
HTC-Pyr	80.83	19.17	88.56	8.45	2.99
